# Validation and Factor Analysis of the Child and Youth Resilience Measure for Indigenous Australian Boarding School Students

**DOI:** 10.3389/fpubh.2018.00299

**Published:** 2018-10-23

**Authors:** Erika Langham, Janya McCalman, Michelle Redman-MacLaren, Ernest Hunter, Mark Wenitong, Amelia Britton, Katrina Rutherford, Vicki Saunders, Michael Ungar, Roxanne Bainbridge

**Affiliations:** ^1^Centre for Indigenous Health Equity Research, School of Health, Medical and Applied Science, Central Queensland University, Cairns, QLD, Australia; ^2^Cairns Institute, James Cook University, Cairns, QLD, Australia; ^3^College of Medicine and Dentistry, James Cook University, Cairns, QLD, Australia; ^4^School of Population Health, University of Queensland, Brisbane, QLD, Australia; ^5^Apunipima Cape York Health Council, Cairns, QLD, Australia; ^6^Aboriginal & Torres Strait Islander Child and Youth Outcomes Project, Australian Research Alliance for Children & Youth, Canberra, ACT, Australia; ^7^Griffith Criminology Institute, Griffith University, Brisbane, QLD, Australia; ^8^Resilience Research Centre, Dalhousie University, Halifax, NS, Canada

**Keywords:** indigenous resilience, adolescents, measurement, cultural adaptation, psychometric assessment, factor analysis, socioecological

## Abstract

**Introduction:** Resilience is a strengths-based construct that is useful for understanding differences in health and wellbeing among youth. There are a range of validated survey instruments available to measure resilience for Aboriginal and Torres Strait Islander (hereafter respectfully Indigenous^1^) youth. However, standard international instruments should only be used if they have been subjected to a rigorous cross-cultural adaptation process and psychometric evaluation in the target population to ensure their validity. The aim of the study was to validate an adapted Child and Youth Resilience Measure (CYRM-28) within a sample of Indigenous Australian boarding school students.

**Method:** The CYRM-28, augmented with an additional 11 site specific items was administered to a purposive sample of Australian Indigenous boarding school students (*n* = 233) as part of the broader T4S survey instrument that captures demographic information and measures resilience, psychological distress and risk, and service usage. Confirmatory factor analysis was undertaken to verify the relationship between the observed variables with the theoretical constructs of the CYRM-28 and previous findings on the factor structure. Cronbach alpha was also calculated to assess the internal consistency of the CYRM-28 within this sample.

**Results:** Survey data were not a good fit for any previously identified models of the CYRM-28, although the inclusion of a site-specific variable improved the overall fit statistics. Two separate scales were confirmed that capture the sources and expressions of resilience for Indigenous Australian boarding school students. This structure is different to previous findings in relation to the CYRM-28, but consistent with conceptualizations of resilience as a dynamic process.

**Conclusions:** The findings are useful in guiding the future use of the CYRM-28 instrument, explorations of Indigenous youth resilience, and for services working with Indigenous youth in out of home care situations. They highlight contextual differences in the measurement of resilience and the importance of validating standard instruments that have been subjected to rigorous cross-cultural adaptation processes. The two scales offer practical guidance to human services working with Indigenous youth on strategies to build and monitor resilience in Indigenous Australian youth and contribute to the emergent understanding of their resilience.

## Introduction

Conceptualizations of resilience have moved beyond the capacity of individuals to bounce back from traumatic situations or setbacks (1). They now encompass a more nuanced understanding of the interaction of both individual assets and the environments people grow and live in, and their connection to culture (2–5). The inclusion of context, the environments of child development and family life, is critical to the examination of resilience, particularly for Indigenous Australian youth for whom the predictors and consequences cannot be assumed from studies of mainstream populations (6). It is well-understood that the experience of any socioecological environment is not homogenous, particularly for youth who experience marginalization or discrimination (7, 8).

There are a range of survey instruments available internationally to measure resilience for both the general population and youth (9–11). However, they are rarely or inconsistently used to inform decision-making in health and education services for Indigenous youth (12) for reasons that include: concerns about risk based questions and their potential impact on students; lack of resourcing for services to conduct intervention-based research; concerns over the capacity to respond to mental health issues raised by the instrument, and; concerns over the appropriateness and relevance of international instruments for utility with Indigenous Australian youth (13). The suitability of international instruments for use with Indigenous Australian youth is also linked to broader concerns of data sovereignty—the production, ownership, and use of Indigenous data—and the cultural appropriateness of using standard measures in Indigenous contexts (14, 15).

A recent review of social and emotional wellbeing (SEWB) instruments for use with Indigenous Australians, recommended that standard international instruments only be used if they had been subjected to a rigorous cross-cultural adaptation process and psychometric evaluation in an Indigenous Australian population to ensure validity (16). This adaptation and evaluation is necessary to address what Walters [(15), p. 46] identifies as the effects of “underpinning racial presumptions” and “realities of resource access” to ensure that measures are able to reflect fundamental differences in ways of being, knowing, and doing between Western and Indigenous peoples (17). Ensuring that measures are appropriate for Indigenous people is also imperative (18).

Prior research in Canada and the United States highlights the importance of accounting for differences in cultural contexts when utilizing standard resilience measures (2, 8, 19, 20). A number of studies that have utilized standard resilience measures with Indigenous youth have included cross-cultural adaptation processes using community participatory research methods to ensure that items reflect local conceptualizations of resilience (21–23). In some instances, these adaptations have resulted in changes to the wording or scoring of the standard scales (22, 24), a process which can have significant effects on the psychometric properties of the scales (25).

## Contextual differences in the factor structure of the CYRM-28

The Child and Youth Resilience Measure (CYRM-28) is an internationally-developed survey instrument to measure child and youth resilience. It has been validated and adapted in a number of cultures and contexts, including Canada, Aotearoa New Zealand, South Africa, and with refugee populations (26–31). Recommendations for any application of the CYRM-28 include the establishment of a community advisory committee to ensure cultural and contextual relevance, including the selection of site specific questions (32). Findings from the application of the instrument in different cultures and contexts show that there are different factor structures for the target populations.

### The CYRM-28 factor structure in canadian youth

In a Canadian validation study conducted by Liebenberg et al. (27) the role of context was found to be important, both in determining the capacity of caregivers, and in mitigating the impact of compromised caregiving. This study used two separate youth samples to initially undertake exploratory factor analysis (EFA), and then confirmatory factor analysis (CFA). They identified 3 subscales in the CYRM-28: individual; relational, and; contextual (27). The individual subscale captured personal skills (5 items); peer support (2 items); and social skills (4 items). The relational subscale included both physical caregiving (2 items) and psychological caregiving (5 items). Finally, the contextual subscale contained those items that reflect a sense of belonging including spirituality (3 items), culture (5 items), and education (2 items). The three factor hypothesized model based on the EFA demonstrated good fit, Tucker Lewis Index (TLI) = 0.957; comparative fit index (CFI) = 0.979; and root mean square error of approximation (RMSEA) = 0.046 (27). The scale was also found to be reliable from the administration to a sub-sample of 53 youths, 3 to 5 weeks apart with the α on subscales ranging from 0.65 to 0.91. Floor and ceiling effects were tested but not detected.

### The CYRM-28 factor structure in aotearoa new zealand youth

The CYRM-28 has also been validated in a Aotearoa New Zealand study (*n* = 593) with 12 to 17 year old at-risk youth currently engaged with the juvenile justice system, child welfare system, mental health system, or supplementary educational programs (29). The CYRM-28 was used in the study because it was specifically developed for vulnerable youth and diverse socio-cultural contexts. Just over one fifth (22%) of the sample were not living with family members as caregivers, many had attended multiple schools (*M* = 5.14, *SD* = 2.89), and had experiences of exclusion, suspension or being held back at school (29). In the Aotearoa New Zealand study, a different factor structure to the previous Canadian validation study was detected. Four factors were identified: individual and relational (family) factors, and two contextual factors—social/cultural and spiritual/community (29). Four items from the individual factor in the Canadian study loaded onto the contextual social/cultural factor: *I know how to behave in different social situations*; *I am given opportunities to become an adult*; *I know where to go to get help*, and; *I have opportunities to develop job skills*. The validity, reliability, internal consistency, factor structure and floor and ceiling effects were examined. The α on subscales ranged from 0.66 to 0.81, no floor or ceiling effects were identified, and, with a sub-sample of 38 youth it was found to be reliable (29). Overall, the findings demonstrated that there were common elements of resilience across cultures, but the way they grouped was different in this context.

### The CYRM-28 factor structure in south african youth

To address differences in previous findings, the factor structure of the CYRM-28 has also been examined in a South African study (30). The study first evaluated models based on prior findings and adapted to the South African context in a sample of 559 youth. It then confirmed the best fitting model in a subsequent sample of 578 youth aged between 12 and 19 years (30). The models tested were based on findings from the Canadian and Aotearoa New Zealand validation studies (27, 29). Alternate versions of the models were also tested. These versions were more representative of African ways of being, particularly in relation to collectivism, holism, and spirituality. One of the alternate models, an adaptation of the Aotearoa New Zealand model with 3 subscales that measured individual resources, family/relational resources, and a contextual composite of social/cultural resources and spiritual/community resources that allowed two items to correlate (*My friends are on my side* and *My friends stand by me during difficult times*), was identified as having the best fit CFI = 0.90, TLI = 0.90; RMSEA = 0.04 (30). The South African study also identified common elements of resilience across cultures, but they grouped differently in this context also.

## Aim

Consistent with recommendations to ensure cultural and contextual relevance (32), the CYRM-28 was cross-culturally adapted for use in our study with Indigenous Australian boarding school students (years 7–12). A six phase participatory action research process was used that resulted in: tailoring the CYRM-28 for the contexts of the distinct environments of boarding school and remote communities; adding 11 site-specific items; addressing relevant wellbeing issues; and delivering it in ways that are appropriate to the literacy levels and age of the students [this phase of the project is detailed at (13)]. That process established both content and face validity for the resultant T4S instrument which incorporates the CYRM-28 (13). However, the wording on a number of items was slightly altered and the easier reading level version of the scale was used (Option 2) with the five point response scale of the standard wording (Option 1), rather than three point response scale of the easier reading version. Changes in wording, and especially response scales can have significant impacts on the psychometrics of a measurement instrument (25). In the present study we assessed the impact of the cross-cultural adaptation process and changes to the instrument by examining the factor structure of the CYRM-28 as part of the process of validating the scale for use with Indigenous Australian boarding school students. Confirming the factor structure for this population was important because previous examinations had demonstrated that whilst there are common elements of resilience across cultures, the way they group can vary. This examination will guide the use of the scale in future investigation of the influence of resilience for the target population, and contributes to the broader understanding of the appropriateness of adapted Western instruments for Australian Indigenous youth.

## Materials and methods

### Background, sample, and administration of survey

Our study engaged a purposive sample of 233 Indigenous Australian secondary school boarding students from remote communities in north Queensland, Australia as part of a 5 year intervention study to increase psychosocial resilience [full details of the study protocol are at (33)]. Students in the study are supported by Education Queensland's Transition Support Service (TSS), a specialist unit that provides assistance to students who leave home to complete secondary schooling through a boarding school placement (34). Ethical clearance was gained from Education Queensland (550/27/1646) and Central Queensland University (H16/01-008).

The mean age of the student participants was 13.42 years (*SD* = 1.7), and 52.4% were female. The surveys were administered during the school term between February and May, 2017, when students were resident at their boarding schools. Consent was given for administration of the survey, firstly by each participants' parent or guardian, and then by individual participants at the time of survey administration. The T4S instrument, which incorporates the CYRM-28 (13) was administered to students either individually or in small groups. Participants completed the survey online using individual devices (iPads).

To minimize situational effects of age, gender, and education level, surveys were administered by the same two researchers (AB and KR) across all sites. In larger sites, some additional assistance was provided by TSS staff under the guidance of the researchers. To address the varying literacy levels and English as a second language needs, students were given the option to have the survey questions read, and as necessary, explained to them by a researcher. Students who completed the survey independently were asked to check with a researcher at the end of each section to ensure understanding before proceeding to the next section. The researchers used consistent language and explanation across all sites. All participants were provided with a brief explanation of the intent of the research, how to indicate their desire to participate, and instructions on how to complete the survey. Surveys were predominantly conducted immediately after school or in the evening at the boarding house. Students were provided with snacks to eat whilst completing the survey but not incentivised through any other means.

The T4S instrument incorporates a range of variables to capture demographic information and measure resilience, psychological distress and risk, and service usage (13). Only measures relevant to the present CFA analysis are outlined below.

#### Demographics

Only participants' age and gender were included in the present analysis.

#### Site specific questions

The CYRM-28 manual recommends the development of site-specific questions to ensure that contextual relevance of the scale for the context of interest (32). Eleven site-specific questions were developed during a rigorous cross-cultural adaptation process (13) to capture the boarding school environment and are shown at Table 1 below. The items are measured on a 5 point Likert Scale, ranging from 1 “None of the time” to 5 “All of the time” for consistency with the CYRM-28 scale.

**Table 1:** Site specific questions for Australian Indigenous boarding students.

		**Context**
1	I worry for my family's safety when I am not at home	School
2	I like the way my school celebrates things	School
3	I feel safe when I am at the boarding house	School
4	I feel safe when I am at school	School
5	I feel that I can speak out and be heard in my class	School
6	I find school work really hard to keep up with	School
7	I feel respected by the teachers at school	School
8	I feel respected by the boarding staff	School
9	I show respect to the teachers at school	School
10	I show respect to the boarding staff	School
11	I know what my language, totem, clan group, or traditional country is	Home

#### CYRM

An adapted version of the CYRM-28 scale (13), was administered to measure participants' resilience. The CYRM-28 has demonstrated high reliability and validity in previous studies, although differences in the factor structure have been identified (11, 27, 29–31). The scale was adapted to suit the unique situation for Indigenous Australian boarding school students as illustrated in Table 2. These adaptations included: changes to wording (including the anchors on the Likert scale); changes to the setting of interest; changes to the sequence of questions, and; the additional 11 situationally specific questions being interspersed with the CYRM-28. All modifications are consistent with the CYRM-28 manual in order to ensure contextual relevance and comprehension among populations experiencing high levels of risk exposure and less familiarity with completing standardized measures (20, 32). The items are measured on a 5 point Likert Scale, ranging from 1 “None of the time” to 5 “All of the time,” with higher scores representing increased resilience (35).

**Table 2:** Adapted CYRM-28 scale for Australian Indigenous boarding school students.

	**Adapted wording**	**Context**
1	I have people I want to be like	Home
2	I share/cooperate with people around me	School
3	Getting an education is important to me	School
4	I know how to behave/act in different situations (such as school, boarding house, home)	School
5	My parent(s) or caregiver(s) watch me closely, they know where I am and what I am doing most of the time	Home
6	My parent(s) or caregiver(s) know a lot about me (like what I do)	Home
7	There is enough to eat at home when I am hungry	Home
8	I try to finish activities that I start	Home
9	Spiritual beliefs are a source of strength to me (eg belief in God, belief in Spirit)	Home
10	I am proud of my Aboriginal and/or Torres Strait Islander heritage (I know where my family comes from or know about my family's history)	Home
11	People think I am fun to be with	School
12	I talk to my family about how I feel (eg. if I am hurt or sad or homesick)	Home
13	When things don't go my way, I can fix it without hurting myself or other people (like hitting others or saying mean things)	Home
14	I feel supported by my friends	Home
15	I know where to go to get help	Home
16	I feel that I belong at my school	School
17	I think my family cares about me when times are hard (like when I am sick or have done something wrong)	Home
18	I think my friends care about me when times are hard (like when I'm sick or have done something wrong)	Home
19	I am treated fairly	School
20	I have chances to show that I am growing up and can do things by myself	School
21	I know what I am good at	School
22	I participate in religious activities (e.g. church youth group)	Home
23	I think it is important to help out in my community	Home
24	I feel safe when I am with my family	Home
25	I have chances to learn skills that will be useful when I am older	School
26	I like the way my family celebrates things (e.g. holidays or learning about my culture)	Home
27	I like the way my community celebrates things (e.g. Festival, Foundation Day)	Home
28	I am a proud Australian	Home

### Analysis

Data were downloaded into Microsoft Excel spreadsheets to facilitate cleaning procedures as recommended by Pallant (36). Any incomplete records that occurred as a result of internet connectivity issues experienced during data collection were identified, checked, and matched against complete records using the participants' school attended, school year, and date of birth. Double entries were identified for three students, and, in these instances the initial entry was kept to avoid any influence of having already completed the survey. Three incomplete records that lacked sufficient responses to calculate a total CYRM-28 score were also removed. A total of 233 records remained for analysis.

Descriptive statistics were examined using SPSS version 24 (IBM, SPSS Inc, Chicago, USA). Internal consistency was assessed using Cronbach's alpha. Floor and ceiling effects were assessed consistent with recommendations of ≤15% of respondent achieving the highest or lowest possible scores (11).

CFA was undertaken due to the strong theoretical basis of the CYRM-28 and previous empirical findings (37, 38). The small sample size also precluded the ability to split the sample and perform EFA first. CFA was conducted using AMOS 24 (IBM, SPSS Inc, Chicago, USA). Because of the sensitivity of the Chi-square test for goodness-of-fit to non-normal distributions, the Comparative Fit Index (CFI), and root mean square error of approximation (RMSEA) were used to assess model fit (39). Akaike Information Criterion (AIC) allowed for model comparison. In isolation the values are meaningless, however, the “best” model is the one with the smallest AIC. CFI values can range from 0 to 1, an acceptable fit to the data is indicated when >0.90, although >0.95 is preferred. RMSEA values ranging from 0.05 to 0.08 indicate an acceptable fit, and values above 0.1 suggest an unsatisfactory fit (39).

Seven models were tested because previous findings have demonstrated that there are common elements of resilience across cultures, but a difference in the way they group between contexts. The seven models were based on: models of the CYRM-28 scale identified in prior factor analysis that had contextual relevance; its application in the current context; a theoretical framework of being, knowing and doing for Indigenous Australians, and; a conceptualization of resilience as both a process and an outcome. The models examined were:

1. The original Canadian CYRM model: The factor structure for this model included 3 subscales that measured individual, relational, and contextual supports. The individual supports subscale included 3 clusters of items: personal skills, social skills, and peer support. The relational supports subscale included 2 clusters of psychological and physical caregiving resources. The contextual supports subscale included 3 clusters: cultural resources, educational resources, and spiritual resources (27).
2. The Aotearoa New Zealand CYRM model: This factor structure included 4 subscales that measured individual resources, family resources, and two contextual factors, social and cultural contexts, and spiritual and community contexts; this is different to the Canadian structure which had only one contextual factor. The second contextual factor contained four items from the individual factor in the Canadian model highlighting the importance of collectivism in this cultural context (29).
3. The South African CYRM model: The factor structure for this model included 3 subscales that measured individual resources, family/relational resources, and a composite of contextual resources that clustered as social/cultural contextual resources and spiritual/community contextual resources (30).
4. Survey contexts model: An innovation in our design is that the T4S instrument divides the CYRM-28 items between the two unique environments that the participants move between, their home community and boarding school. The first section of questions is situated in the school and boarding environment, stating to participants that, “These questions ask about your experience at school and in the boarding house.” The second section of questions is situated at home and in their community, “These questions ask about you, your family, and your home community” (13). To address the influence of context, the survey contexts model hypothesized two factors, one capturing those items measuring aspects of resilience relating to home and one for boarding school.
5. Being, knowing, and doing theoretical model: In the absence of an Indigenous model or conceptualization of resilience, we hypothesized a model based on theories of Indigenous ways of being, knowing, and doing (17) and with the approach taken in the South African study (30). Items from the CYRM-28 scale were categorized (being, knowing, and doing) using an amended Delphi technique led by an Indigenous researcher (RB) and including three other researchers familiar with the CYRM-28 scale and the research context (JM, MRM, EL).
6. Capacity, process, and outcome: Based on previous conceptualizations of resilience as a dynamic process (8, 40, 41) and consistent findings on empowerment in Indigenous Australian women (42, 43), we classified the items from the CYRM-28 as being either sources (capacity) or expressions (outcome) of the participant's resilience in a 2 factor scale. Items classified as a source of resilience were: externally driven; collective; such that students were placed in a passive relationship with them, and; beyond student's control. In contrast, items classified as expressions of resilience were: internally driven, individually based; such that the student took an active stance; and; mostly in the control of the student.
7. CYRM-12: We also examined the fit of the CYRM-12 (a subset of the CYRM-28) because of the absence of items that pertain to physical caregiving by the participants' primary caregiver(s). This has contextual relevance because responses to questions regarding caregiving can be influenced by the participant's locations at boarding schools as well as the ongoing impact of colonial policies such as family separations and placing children in out of home care. The CYRM-12 is a single factor scale.

## Results

### Descriptive analysis

More than half the participants were aged 13 years or younger. Gender balance differed by age, with a higher proportion of females up to the age of 14 years, but a higher proportion of males aged older than 14 years. A full breakdown of participant gender and age is shown below at Table 3.

**Table 3:** Participant age and gender count and (Percentage).

**Age(years)**	**Male**	**Female**	**Total**
11	12 (5.2)	26 (11.2)	39 (16.7)
12	19 (8.2)	29 (12.4)	48 (20.6)
13	21 (9.0)	20 (8.6)	41 (17.6)
14	16 (6.9)	20 (8.6)	37 (15.9)
15	19 (8.2)	11 (4.7)	30 (12.9)
16	21 (9.0)	13 (5.6)	35 (15.0)
17	3 (1.3)	3 (1.3)	6 (2.6)
Total	111 (47.6)	122 (52.4)	233

Cronbach's alpha for the CYRM-28 items was 0.838. Whilst there were some negative correlations between a few items, the removal of any items would not improve the alpha. An expanded resilience scale that included the 11 additional site-specific items (Table 1), had an α of 0.876. This level improved to 0.881 if the item *I find school work really hard to keep up with* was removed. This result is unsurprising because it is the only question that is negatively worded. No floor effects were identified within the sample, but every item except *I find school work really hard to keep up with* demonstrated ceiling effects outside recommended guidelines (11).

### Factor analysis

The hypothesized models for the adapted version of the CYRM-28 items included: three models based on previous empirical findings (Canadian model M1; the Aotearoa New Zealand model M2; the South African model M3); a model based on the contextual structure of the survey (M4); a model theorized to capture Indigenous concepts of resilience (M5), and; a model theorized to capture sources and expressions of resilience (M6) and the CYRM-12 (M7).

As shown at Table 4 below, the data were not a good fit for any of the models from the three previous validation study findings (M1, M2, or M3), the survey contexts model (M4), or the hypothesized model based on Indigenous concepts of resilience (M5). An examination of the fit indices for all these models identified problems with the item *I feel that I belong at my school*. A retroductive (44) consideration of this item suggested that belonging may represent a higher order, or aspirational, connection between the student and the school that would take time to develop. We hypothesized that connection to school could exist as a hierarchy and feeling safe at school would be a necessary step before a student could feel that they belonged. Given the large proportion of younger students and students who had moved high schools in the sample, and who were therefore new to their school, the item was replaced initially with one of the site specific items *I feel safe when I am at school*, and then *I feel safe when I am at the boarding house* to examine contextual differences, and the importance of the boarding house in the student's lives. The use of the replacement variable *I feel safe when I am at the boarding house*, improved the model fit for all models, as shown at Table 4 (replacement variable). However, the overall fit of the data to the models was still not good. The CYRM-12 also did not have good fit indices, despite the contextual relevance.

**Table 4:** Goodness-of-fit for CYRM-28, CYRM-28 with replacement variable, and CYRM-12.

**Models**		**χ^2^ (*df*)**	**CFI**	**RMSEA**	**AIC**
**CYRM-28**					
1	Canadian Model	600.229 (339)	0.775	0.057	790.229
2	*Aotearoa* New Zealand Model	553.931 (344)	0.811	0.051	733.931
3	South African Model	602.784 (345)	0.778	0.056	780.784
4	Survey context	532.914 (336)	0.828	0.050	728.914
5	Being Knowing Doing	633.564 (399)	0.815	0.050	825.564
**CYRM-28 (REPLACEMENT VARIABLE)**					
1	Canadian	577.253 (339)	0.792	0.055	767.253
2	*Aotearoa* New Zealand	538.709 (344)	0.822	0.049	718.709
3	South African	582.152 (345)	0.793	0.054	760.152
4	Survey context	518.358 (336)	0.841	0.048	714.358
5	Being Knowing Doing	639.348 (344)	0.740	0.061	819.348
**SOURCES AND EXPRESSIONS OF RESILIENCE**					
	Sources of resilience	133.363 (88) *p* = 0.001	0.922	0.047	197.363
	Expressions of resilience	75.071 (65) *p* = 0.184	0.960	0.029	127.071
**CYRM 12**					
CYRM12		95.429 (51)	0.818	0.061	149.429

*df, degrees of freedom; CFI, comparative fit index; RMSEA, Root mean square error of approximation; AIC, Akaike Information Criterion*.

A sources and expressions of resilience model was initially conceptualized and tested as a single model with sources and expressions of resilience as two separate factors. They were also tested as independent models of sources or expressions of resilience. When tested as independent models, they each provided a good fit for the data. The item *I know what I am good at*, proved problematic as either a source or expression of resilience and was not included in either model. Based on the results for the other models, the item *I belong at school* was again replaced with a site specific item *I feel safe when I am at the boarding house* which improved model fit. Additional site specific items from the survey were then also considered for inclusion. The item *I worry for my family's safety when I am not there*, improved the model fit for the expressions of resilience model.

**Figure 1:**
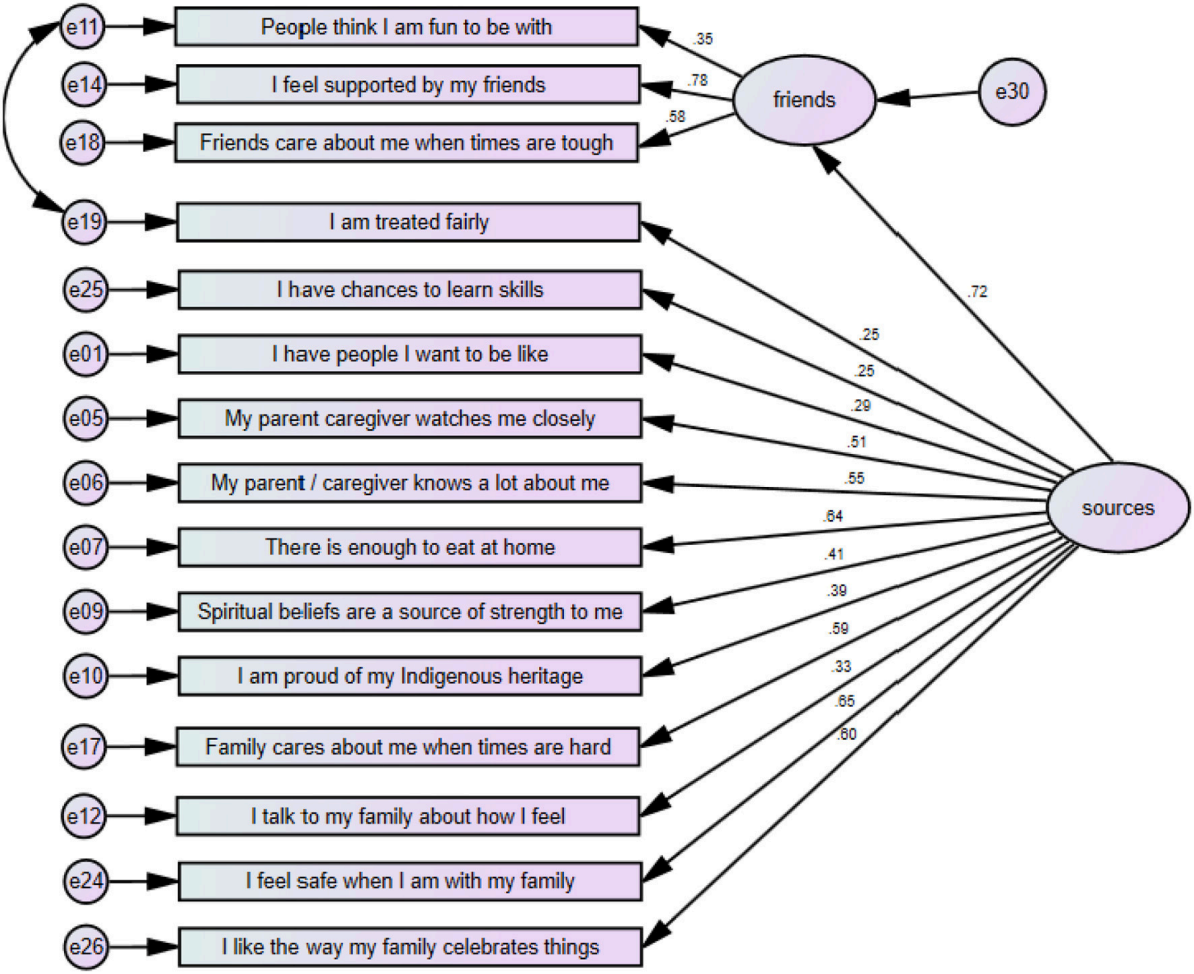
Sources of Resilience Model including standardized item loadings.

Overall fit for the sources of resilience model, illustrated at Figure 1 below, was χ^2^ 133.4 (88) *p* = 0.01, CFI = 922, RMSEA = 0.047. The error terms for two items, *People think I am fun to be with*, and *I am treated fairly*, were allowed to co-vary based on theoretical considerations and improved model fit. The model was tested for structural invariance, firstly between genders, and then between age groups of younger (<15 years) and older (15 years and above) students. There were differences between genders at the model level. An examination of path differences highlighted that being treated fairly, loaded more for males, whilst having closeness to parents (caregivers), pride in their Indigenous culture, and supportive friends, loaded more for females. *I have people I want to be like*, had the biggest difference between genders. Similar loadings were noted between genders for *feeling safe with family*.

An examination of path differences between age groups also identified important differences for people the students wanted to be like; this loaded at 0.18 for the younger group, and 0.46 for the older group. A number of other items had higher loadings for older students, including those items concerned with friends, family celebrations of culture, and spirituality. Factors with higher loadings for younger students were *being treated fairly* and *talking to their family about how they feel*.

**Figure 2:**
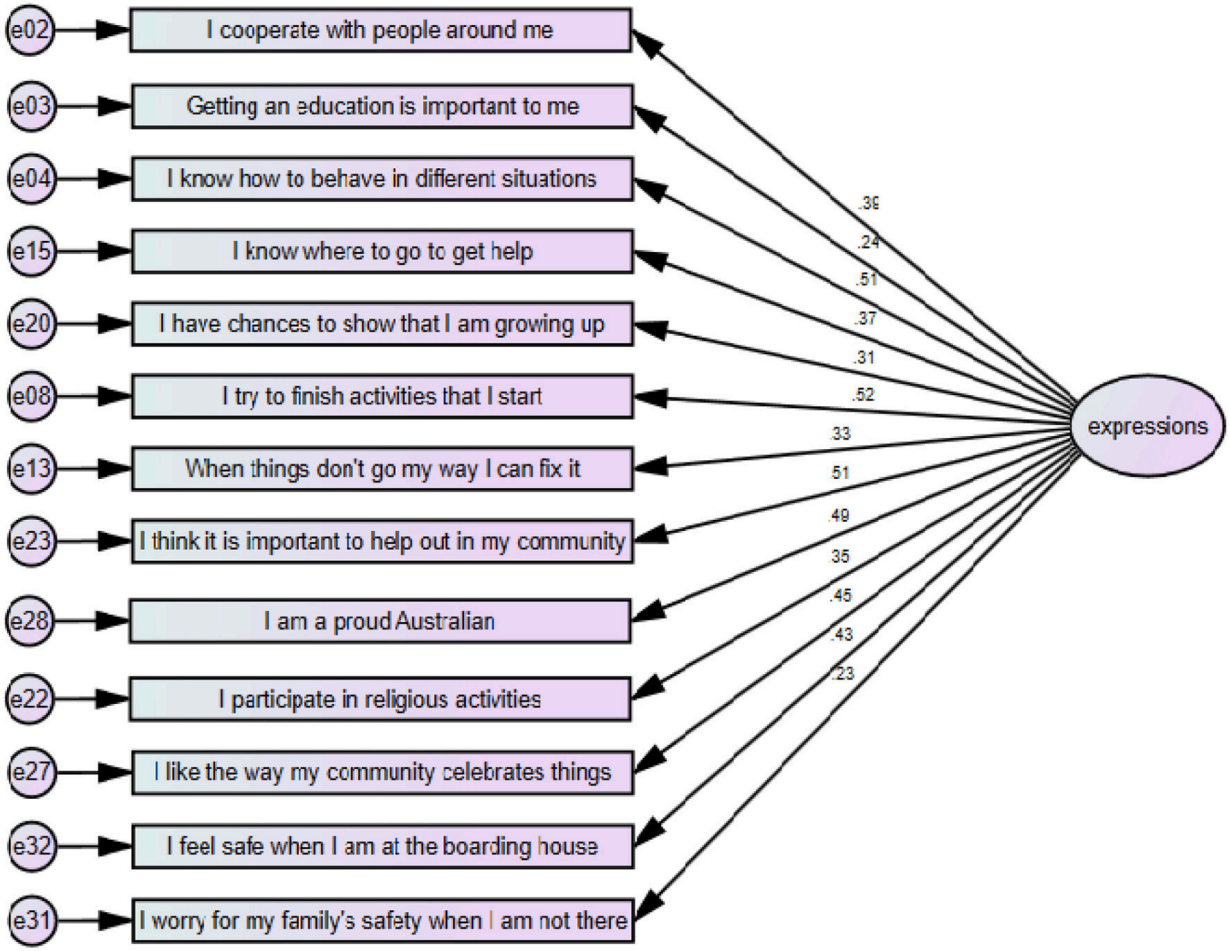
Expressions of Resilience including standardized item loadings.

The expressions of resilience model, illustrated at Figure 2, was also a good fit for the data χ^2^ 75.1(65) *p* = 0.184, CFI = 0.960, RMSEA = 0.026. Testing for structural invariance for expressions of resilience between both gender and younger or older age groups found that there were no differences at a model level.

## Discussion

In this study, we examined the factor structure of the CYRM-28 in a sample of Australian Indigenous secondary boarding school students as part of a broader validation process and to specifically assess the impact of contextual and cross-cultural adaptation processes which amended a standard measurement instrument. Previous studies examining the factor structure of the CYRM-28 across samples in different countries demonstrated that whilst there are common elements of resilience across different populations, the way items grouped was different between contexts (27–31). These previous examinations of the factor structure of resilience highlighted aspects of relevance the our study: the importance of parent (caregiver) capacity; a sample of vulnerable youth; and more collective ways of being (27, 29, 30). Despite the noted commonalities, our data were not a good fit for any of the models identified in those studies. Hypothesized models based on models of Indigenous theoretical concepts of resilience (17), and the context of the study (13) were also tested, as well as the CYRM-12. Again, the data were not a good fit for these alternate models. Subsequent analysis revealed two distinct scales that captured the sources and expressions of resilience for Australian Indigenous boarding students, although not all of the CYRM-28 items were retained, and two site specific contextual items were included to improve model fit. The findings are consistent with theory developed through a prior study that found the resilience and empowerment of Indigenous women in Australia is dynamic and non-linear (42, 43), and broader theories of resilience as a process (8, 40, 41).

**Figure 3:**
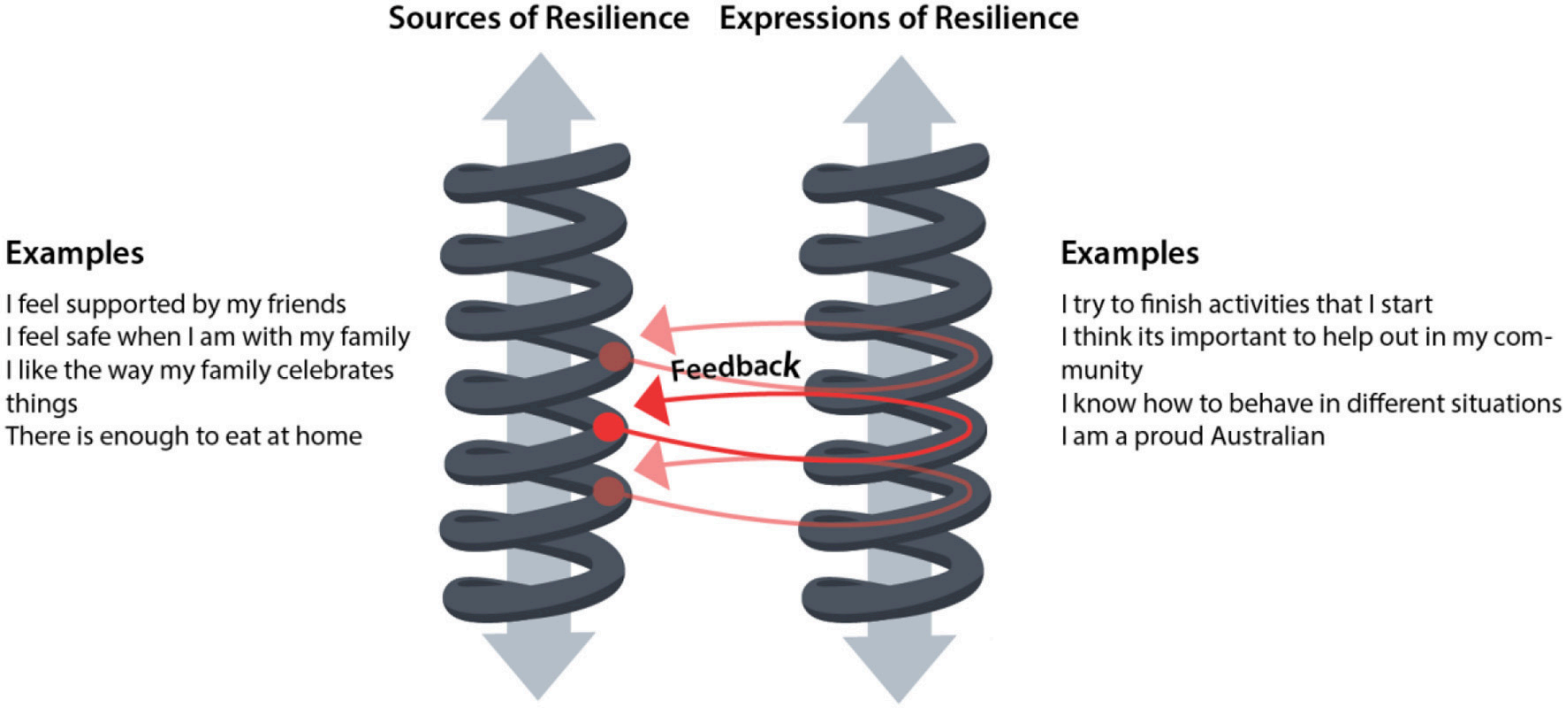
Hypothesized dynamic interaction of sources and expressions of resilience.

We theorized that the two scales, sources and expressions of resilience, were separate but connected through feedback loops. Theories of being, knowing, and doing (17), allowed us to reconceptualize resilience as something that was sourced through being and knowing, but was expressed through doing. Students must have resilience (sources) to express it. For example, knowing that friends care, support them, and think they are fun is a source of resilience for a student. Cooperating with their friends is an expression of resilience; it draws on knowledge that they are cared for and valued. Additionally, expressions of resilience that are successfully repeated over time could become, or contribute to sources of students' resilience. For example, students who can cooperate within their peer groups, will feel the support and care of friends. This provides a feedback loop that strengthens the source of resilience (thus increasing their resilience) and supports them in expressing it more through cooperation in the future. This dynamic process is illustrated below at Figure 3. These expressions and feedback loops could exist as multiple micro-events across time, creating incremental changes in resilience in a positive or negative direction. However, they might also include single events that could have more profound effects. For example, a student who has not had chances to learn skills that would help them adapt to the boarding school environment might express lower levels of resilience because they do not know the appropriate behavior in a particular situation on commencing school. If the outcome of this expression is a more significant negative feedback, such as getting in trouble or being ridiculed by their peers, then this will have a more pronounced negative effect on their level of resilience.

In environments of changing sources of resilience (contingent support), the feedback loop reflects the capacity of individuals to be connected, and to shift that connection. Wexler et al. (45) working with Alaskan Native youth, identified the importance of connection as a resilience strategy, and also the practice of cultivating relatedness, that is, developing relationships that took on qualities of kinship. This influence of relatedness, in our findings especially to friends, is consistent with this.

Sources of the students' resilience lie within the homes and communities to which they belong. This is particularly so for their connection or relatedness to others, and is consistent with previous findings from research with Indigenous Australian and Māori boarding students (46), and Alaskan Native youth that identified this as a resilience strategy (45). The items that make up the sources of resilience for these students share four important characteristics. Items classified as a source of resilience were: externally driven; collective; such that students were placed in a passive relationship with them, and; beyond student's control. They are also consistent with findings from the Canadian validation study (27) that identified the importance of the capacity of caregivers and the role of compensatory factors in determining youth's resilience. The collectivist nature of the sources of resilience is consistent with Indigenous Australian ways of being (17).

Pride in Indigenous heritage was a source of resilience for the students, but there was no item within the CYRM-28 which captured cultural expression at an individual level. Students' sources of resilience also draw on their relationships with family, friends, community, and those who care for them in settings away from home. This is consistent with the embeddedness of Indigenous Australian cultures' in kinship and collectivism and previous findings on the importance of connection and relatedness as a resilience strategy for Indigenous youth (45). Items captured relatedness to parents (caregivers), family, and friends. The nature of Indigenous youth peer groups and relationships means that friends often include siblings, cousins, and extended family or clan.

Gender and age differences between some sources of resilience items is worth highlighting. The importance of the considering gender, age, and other social determinants of health in developing resilience in youth has been previously identified (41). The differences in the influence of family and friends as sources of resilience between age groups suggests that there is a deepening or emotional maturing that occurs. For the younger age group, the more active aspects of this relationship, such as talking to their family about how they are feeling, loaded more strongly as a source of resilience. Males and younger students' sources of resilience were more impacted by being treated fairly by those around them. The older age group and female students drew more on the intangible feelings of closeness to parents (caregivers), family and friends. We found that being treated fairly was more influential for males, whilst closeness to parents (caregivers), pride in culture, and supportive friends, having people they want to be like, and feeling safe with their family was more influential for females. For students over 15 years of age (mostly in their last 2 years of high school), having people they want to be like, supportive and caring friends, and family celebrations of culture also held more influence.

Items that make up the expressions of students' resilience differ from the sources in the same four characteristics. Expressions of resilience are: internally driven, individually based; such that the student took an active stance; and; mostly in the control of the student. Expressions can manifest in a positive or negative way; for example, cooperating or not cooperating with others, finishing or not finishing activities. Items that reflected students' relatedness to structures were mostly expressions of resilience.

### Implications

The findings are useful in guiding the future use of the CYRM-28 instrument, explorations of resilience in Indigenous youth, and for human services working with Indigenous youth. The importance of forming a community advisory committee to ensure cultural and contextual relevance, including the selection of site-specific questions when using resilience measures is supported by our findings. Walter (15) argues that statistical measures are not neutral, and that the influence of social and cultural artifacts must be considered. This is evidenced in the item that refers to students “belonging” to school. The term has an intimate and spiritual meaning within Indigenous Australian communities and their relationships to country. Additionally, although the word belonging can convey a sense of relatedness and affinity, it can also have patriarchal and possessive undertones given the colonial experiences of Indigenous Australians (47).

For services working with Indigenous youth, including education, health, and welfare services, the scales can be used to identify potential opportunities for developing or monitoring resilience. The importance of the socio-ecological environment as a source of resilience has been clearly identified in previous research (2–5), and our findings demonstrate the importance of fostering connection and skills in developing relatedness for Indigenous Australian youth. Importantly, these settings must consider the role of institutional factors such as cultural competencies in supporting the strengthening of young people's resilience. The expressions of the resilience scale offers an effective guide for determining individual capacity at a point in time.

### Limitations and future research

This study engaged a purposive sample of students from north Queensland communities who attend a limited number of boarding schools, and who are supported by a single state government service in their transitions to schools. As such, the findings should not be generalized to all Indigenous Australian students, or all Indigenous Australian youth. Differences in the history and situation of the students' home communities, school environments, and experiences of support will have an influence on the findings. Social desirability bias might have influenced responses. Surveys were administered in the school environment by researchers who students may associate with the government support service. The potential influence of this, especially on the item *Getting an education is important to me*, was balanced against the importance of research staff having an established relationship with the students to ensure student safety in the research process. The more general influence could be on questions relating to parent or caregiver(s) capacity, such as *There is enough to eat at home when I am hungry*. The persisting effects of family-disruptive colonial policies and contemporary elevated rates of removal of Indigenous children to out-of-home care may have influenced students' to respond positively to these questions. Furthermore, the innate complexity of items such as these is compounded in this cultural context where certain practices that supported group cohesion and individual resilience in traditional (historical) settings—such as demand sharing—may, on the one hand, be considered to reflect culturally appropriate expressions of resilience (8), but may also have different if not opposing consequences in the very different contexts of contemporary remote Indigenous communities within a wider, globalized society (48–50).

Overall, we cannot determine whether our findings are the result of Indigenous Australian concepts of resilience, the context of boarding school attendance, or a combination of both. Further research is recommended that compares four distinct groups: Indigenous day students; Indigenous boarding students; non-Indigenous day students; and non-Indigenous boarding students to determine the role cultural and contextual factors play. However, we note that there might not be a single concept of resilience between Indigenous Australian youth, who have varied and unique cultural beliefs and practices, histories, and experiences of colonization that would be of influence. Furthermore, we need to improve our understanding of the sources of student's resilience; especially the differences that gender and age play in this and a consideration of institutional factors. This might be better addressed through constructivist methods, especially those that prioritize students' voices to share their understanding and experiences.

## Conclusion

This study was undertaken as part of a broader validation process of a standard resilience measure to assess the impact of a process of contextual and cultural adaptation for use with a group of Indigenous Australian secondary boarding school students. The adaptation process ensured that the instrument was a good reflection of resilience for the target population but may have affected the psychometric properties of the instrument. Our findings were inconsistent with previous studies using the same instrument. Two separate scales with good internal consistency were confirmed that captured the sources and expressions of resilience for Indigenous Australian boarding school students and highlighted the utility of including context specific measures to strengthen standard measures of resilience. Our findings suggest that resilience for this population should be seen as both unique and as sharing some qualities with other conceptualizations of resilience from around the world. This study opens the door to justify further local explorations of positive development that reflect local constructions of doing well under conditions of adversity. Our findings provide guidance for human services working with Indigenous youth on opportunities to improve students' resilience by addressing factors identified as sources of resilience, and to monitor wellbeing through assessing the expressions of resilience.

## Ethics statement

This study was carried out in accordance with the recommendations of National Statement on Ethical Conduct in Human Research (2007). The protocol was approved by the CQUniversity Human Research Ethics Committee and the Education Queensland Ethics Committee. All subjects gave written informed consent in accordance with the Declaration of Helsinki.

## Author contributions

EL, RB, JM, and MR-M contributed to the conceptualization of the study. MR-M, AB, and KR contributed to the acquisition of the data. EL conducted the analysis of the data. EL was primarily responsible for writing the draft. JM, RB, and EH contributed to the draft. EL, JM, RB, MR-M, MW, EH, VS, and MU contributed to the interpretation of the data. All authors contributed feedback, and critically edited the draft. All authors have approved the final version for submission and agree to be accountable for all aspects of this work.

### Conflict of interest statement

Although RB and JM are editors of this special edition, they were not involved in the peer-review or editing of this paper. The remaining authors declare that the research was conducted in the absence of any commercial or financial relationships that could be construed as a potential conflict of interest. The reviewer TAC and handling editor declared their shared affiliation at time of review.
